# Open-Label Placebo Interventions With Drinking Water and Their Influence on Perceived Physical and Mental Well-Being

**DOI:** 10.3389/fpsyg.2021.658275

**Published:** 2021-12-06

**Authors:** Marco Rathschlag, Stefanie Klatt

**Affiliations:** ^1^Institute of Exercise Training and Sport Informatics, German Sport University Cologne, Cologne, Germany; ^2^Institute of Sports Science, University of Rostock, Rostock, Germany; ^3^School of Sport and Health Sciences, University of Brighton, Brighton, United Kingdom

**Keywords:** open-label placebo, dehydration, acute recovery, physical performance capability, mental performance capability

## Abstract

In recent years, the postulation that deception is necessary for placebos to have an effect on pain relief or increased well-being has come into question. Latest studies have shown that an openly administered mock drug works just as well as a deceptively administered placebo on certain complaints. This open-label placebo effect has primarily been used in the area of pain treatment so far. This study is the first to examine the effect of such placebos on healthy individuals with the use of drinking water. In two experiments, participants were required to use certain specified water bottles for their daily drinking water consumption. At the beginning of Experiment 1, all participants (*N* = 68) received one bottle of water, which they were asked to refill themselves each day during a 2-week intervention period. In Experiment 2, participants (*N* = 75) received a new sealed water bottle every day. In both experiments, participants were randomly assigned to one of four groups: no treatment (control group CG), open-label placebo without rationale (OPR^–^), open-label placebo with rationale (OPR^+^), and open-label placebo with additional rationale in a suggested relaxed state (group OPR^++^). We conducted baseline and post-treatment measurements of the subjective perceived physical and mental well-being of the participants. In Experiment 1, only the OPR^++^ group reported enhanced vitality at the post-treatment level compared to the other groups. In Experiment 2, post-treatment measurements showed improvements for the OPR^++^ group in the Physical Performance Capability, Mental Performance Capability, Emotional Balance, Overall Recovery, Negative Emotional State, and Overall Stress categories compared to the other groups. Our results support the idea that placebos with an additional rationale in a suggestive relaxed state are more effective than with just a rationale in a normal state. Furthermore, our study shows the tendency that OLP^++^ in the form of water with health claims may be more effective when the water is given in several sealed bottles separately than in one sealed but refillable bottle.

## Introduction

Placebos and their effects on health and well-being have been a fascinating research field for years. In areas like treatment and diagnostics, placebos have been used frequently with a variety of positive outcomes. Moerman (2013, p. 381) defines a placebo as an “inert substance (a sugar or starch pill, a saline injection) […] that doesn’t do anything, [and] has no effect on human physiology.” This definition is often quite controversial as many researchers find it questionable (Brody, 2000; Howick, 2017) because a sugar pill, for example, is not inert for patients of diabetes (Annoni and Blease, 2018).

A systematic review by Charlesworth et al. (2017) concluded that many patients experience positive effects even when they know about missing active substance in the administered medication. There are many cases where these so-called open-label placebos (OLPs) have led to symptom reduction even without deception. For instance, OLPs have led to alleviation of symptoms in irritable bowel syndrome (Kaptchuk et al., 2010), reduction of lower back pain (Carvalho et al., 2016), and episodic migraines (Kam-Hansen et al., 2014), improvements to attention deficit hyperactivity disorders (Sandler and Bodfish, 2008; Kaptchuk et al., 2010; Sandler et al., 2010), or even allergic symptoms (Schaefer et al., 2016). Research has also pointed out that while administering OLPs, the instructions and the rationale are very important factors (Von Wernsdorff et al., 2021). What this means is that the OLP administrator explains the inactive nature of the placebo to the subject, followed by positive statements, e.g., about the efficacy and benefits of such treatment. Such rationale may increase positive expectations, which can lead to overall positive effects from the OLP.

Another interesting finding in the past has been emphasized by Locher et al. (2017) who found that OLPs, combined with a plausible rationale (OPR^+^), are more effective than without a rationale (OPR^–^). The rationale can, therefore, affect the expectations of the outcome and influence the extent of the placebo effects (Locher et al., 2018). Furthermore, subjects being administered with OLP with extended information—in comparison to those without the extended information—showed more positive results in mental sum scores of the quality-of-life questionnaires. Locher et al. (2017), however, did not find any differences between the OPR^+^ and the deceptive placebo group. Therefore, the authors questioned the necessity of concealment in placebo administration at all.

This contention that the rationale is one of the key factors in the positive expectations with regard to OLP is supported by the mindset matter literature (e.g., Crum et al., 2017; Zion and Crum, 2018). Crum and Langer (2007), for example, investigated whether the relationship between exercise and health is moderated by the mindsets of the participants. Female room attendants who were told that their job is a good exercise showed significant improvements 4 weeks after the intervention in several health variables compared to the control group, which was not given this information.

Due to associative learning processes, prior treatment experiences usually affect the expectations of the patients more than just verbal information (Voudouris et al., 1985, 1990). It is assumed that if pain, for example, is induced by behavioral conditioning processes, the effect of this unconscious learning experience is more effective in reducing pain than just verbal information. It is then conceivable that OLP effects are traced back to conscious expectations, as this is frequently the explanation on why placebos work. When a doctor promises that an ointment relieves pain, it might actually alleviate the symptoms of pain in the patient based on the belief that the ointment is, in fact, effective. Sometimes the belief in a possible quick recovery is enough to reduce the intensity of pain in patients. Similarly, much like deceptive placebos, OLPs are usually combined with positive recommendations (Charlesworth et al., 2017), making it plausible that the same mechanisms are responsible for the effects.

The importance of conscious expectations is further consolidated by neurobiological studies in the field. In the past, imaging techniques such as Positron Emission Tomography or Magnetic Resonance Imaging have been used to observe the effects that placebos have on neurobiological processes (e.g., Petrovic et al., 2002; Lorenz et al., 2003; Wager et al., 2004). Research shows that hope activates certain regions of the brain which help in recovery. In patients with Parkinson’s disease, motor function improved with placebos and they exhibited a temporary increase in the levels of dopamine. Subsequent studies have shown similar results with patients experiencing pain, indicating an increased release of endogenous opiates (e.g., Medoff and Colloca, 2015). Other studies exhibited an increase in the bonding hormone, oxytocin, indicating confidence in the therapy process (e.g., Ito et al., 2019). Despite the lack of the same level of conscious expectations as deceptive placebos, subjects receiving OLPs with positive recommendations largely exhibit the same mechanisms. Evidence suggests that OLPs combined with an expectation of therapeutic benefit may, therefore, improve healthcare outcomes without the ethical worries inherent in deceptive placebos (Petkovic et al., 2015).

So far, the effect of OLP has primarily been investigated with the administration of medication. While the resulting positive effects, such as the reduction of symptoms of irritable bowel syndrome, depression, attention deficit hyperactivity disorder, chronic lower back pain, and allergic rhinitis, are well documented (e.g., Kaptchuk et al., 2010; Sandler et al., 2010; Kelley et al., 2012; Carvalho et al., 2016; Schaefer et al., 2016; Hoenemeyer et al., 2018), we have deliberately linked the effect of OLP to a different thematic focus in this study.

Our primary goal was to investigate whether a positive effect can be caused by the so-called OLP in combination with a commonly used product of daily consumption by people without any pre-existing health problems. More precisely, we examined whether subjects could achieve a better well-being through the use of OLPs. According to the definition given by the WHO, mental health is a state of well-being in which individuals realize their own abilities, can cope with the normal stresses of life, can work productively and fruitfully, and are able to make a contribution to their community (World Health Organization [WHO], 2003). Positive mental health can be conceptualized as a subjective and perceptive sense of well-being which also affects the physiological health of individuals.

The unit of analysis chosen for our investigations in Experiment 1 and Experiment 2 was drinking water as it is a neutral element consumed by everyone on a regular basis. Participants were randomly assigned to one of the four groups: no treatment (control group CG), open-label placebo without rationale (OPR^–^ group), open-label placebo with rationale (OPR^+^ group), and open-label placebo with additional rationale in a suggested relaxed state (OPR^++^ group). The following hypotheses were tested based on our experiments comparing the responses of participants to various categories of the water bottles: First, the well-being of the participants is enhanced after the application of our water with a rationale (OPR^+^, OPR^++^) compared to OPR^–^ and when the participants do not receive any treatment (CG). Second, with an additional rationale through an audio file (OPR^++^: awake plus trance state), we can increase the positive effect on the well-being of the participants in comparison to adding just a rationale in an awake state (OPR^+^).

## Experiment 1

### Method

#### Participants

To calculate sample size requirements, the G^∗^Power 3.1, Düsseldorf, Germany (Faul et al., 2009) was used. The power analyses indicated that a sample size of at least 56 participants (16 participants per group) would result in a power of 0.95 (α-level = 0.05, *f* = 0.25). In order to adjust for any absences due to sickness—especially due to the COVID-19 pandemic in 2020—we decided to request 80 subjects in advance. Finally, data was recorded from 68 subjects (49 males, 19 females) aged 18–43 years (*M*_age_ = 22.04 years, *SD* = 3.73 years). Informed consent was obtained from each of the participants prior to testing in line with the Declaration of Helsinki, and ethical approval was obtained from the lead institution (number 171/2020).

#### Procedure

Participants were randomly assigned to the CG (*n* = 17), the OPR^–^ (*n* = 17), the OPR^+^ (*n* = 17), and the OPR^++^ (*n* = 17) group. We used a 2 (Time of measurement: T1 vs. T2) × 4 (Group: CG vs. OPR^–^ vs. OPR^+^ vs. OPR^++^) design. On arrival, all the participants filled out questionnaires about their subjective physical and mental well-being (for more details, see section “Measures and Questionnaires”). After baseline measurements (T1), the treatment phase started (2 weeks). Participants in the CG did not receive any treatment or were made aware of the purpose of the study and were told that they were the control group (CG; they were only informed about the study purpose after its completion).

All the participants in the other groups (OPR^–^, OPR^+^, and OPR^++^) were given a sealed water bottle (1 L) with a different rationale for each of the groups. In the OPR^–^ group, the participants were told: “You are receiving this placebo. This water bottle does not contain any pharmacological substance. It is just water. Please refill this particular bottle with any kind of still water during the two-week intervention period as often as possible.” No additional information regarding placebo mechanism was provided to this group and the bottles were not labeled.

In the OPR^+^ group as well, the participants were told that their bottle did not contain any pharmacological substances and was, therefore, a placebo. However, in addition to this, the investigator told them that the placebo effect is usually very powerful, even if people know that it is a placebo (OLP) and gave some examples from previous studies where participants benefited from a placebo. The investigator also mentioned that placebos can activate physical and mental well-being and a positive attitude can be helpful in such a process. All the participants were told: “We want to use bottles with water as a placebo which might be helpful to enhance your subjective physical and mental well-being.”

To help the participants to remember this placebo effect with physical and mental well-being benefits, we labeled their bottles with the slogan “Imagine I am Health.”

In the OPR^++^ group, the participants received the same information and the same labeled bottles as the OPR^+^ group. However, this group also received an audio file of 15.48-min duration to download. The audio file contained the voice of the speaker combined with a piece of relaxing music (“Mountains of Peace” by Andreas Hoegel). It was designed just for this study and used two typical aspects often used in meditations or hypnotic interventions: relaxation and imagination. The first part of the audio file was a relaxation exercise where the participants were instructed by the speaker to relax in their own way. The participants were led by the speaker to first focus on their breath and imagine that they are going into a deeper state of relaxation with every breath they take. Afterward, the speaker counted, very slowly, backward from 10 to 0 where the participants were asked to imagine going deeper into this relaxed state. In the second part, the rationale from the investigator that people can benefit very strongly from a placebo, also when it is an OLP, was reiterated. The participants were then asked to visualize how a complete state of health and well-being could feel in their body. This health suggestion was anchored with the bottle, thus, connecting this state of complete health and well-being with the consumption of the water from the labeled bottle. The participants were asked to use this audio file as often as possible during the experiment.

After the treatment phase of 2 weeks, the participants filled out the questionnaires about their subjective physical and mental well-being again. All the participants were debriefed about the concept of this experiment after the study conclusion.

#### Measures and Questionnaires

First, the assessment of the participants contained demographic variables (age, sex, nationality, job status). Thereafter, they responded to the specific Questionnaire for Assessing Subjective Physical Well-Being (“*Fragebogen zur Erfassung des Wohlbefindens*” or FEW-16 for short; cf. Kolip and Schmidt, 1999) and the Acute Recovery and Stress Scale (ARSS; Hitzschke et al., 2016; Kellmann et al., 2016). The same questionnaires were completed at T1 (baseline measurement) and T2 (after the 2-week intervention phase).

#### Questionnaire for Assessing Subjective Physical Well-Being

The FEW-16 questionnaire (cf. Kolip and Schmidt, 1999) is a questionnaire for assessing well-being. Using different samples, e.g., patient populations from a rehabilitation center and sample population of healthy university students, the FEW-16 questionnaire has been validated in previous research showing good reliability and internal consistency. The internal consistency of the overall scale is 0.92, while Cronbach’s alpha for the subscales is between 0.82 and 0.90 (cf. Albani et al., 2006).

The questionnaire consists of 16 items and contains four items for each of the four subscales: *Resilience*, *Ability to Enjoy*, *Vitality*, and *Inner Peace*. Participants usually answer items using a 6-point Likert scale ranging from “fully applies” (yielding 5 points) to “does not apply at all” (yielding 0 points). Low scores indicate poor physical well-being, and higher scores indicate a better outcome. In this experiment, the subscale values were calculated as usual, as the mean of the values for each of the subscale item. The mean of the four subscale values indicated the total score of the participants (cf. Tahiroviæ et al., 2015).

#### Acute Recovery and Stress Scale

The Acute Recovery and Stress Scale (ARSS) questionnaire (Hitzschke et al., 2016; Kellmann et al., 2016) consists of four recovery and four stress scales representing physical, mental, emotional, and overall dimensions. A list of 32 adjectives/expressions (each describing a different state of recovery and stress, e.g., “rested”, “tired”) are categorized under the eight scales. Each expression is answered on a 7-point Likert-type scale ranging from 0 (does not apply at all) to 6 (fully applies). Four adjectives are grouped as the mean score so that the eight scales can be calculated representing the *Recovery* dimension with *Physical Performance Capability*, *Mental Performance Capability*, *Emotional Balance*, *Overall Recovery*, and the *Stress* dimension with *Muscular Stress*, *Lack of Activation*, *Negative Emotional State*, and *Overall Stress*. The eight scales of the ARSS scores have been found to possess good internal consistency using Cronbach’s alpha ranging from 0.76 to 0.90 (Hitzschke et al., 2016).

#### Use of Water Bottles and Audio File

To investigate how often the participants of the OPR^–^, OPR^+^, and OPR^++^ groups used the assigned water bottles, Likert scales were used. Participants (except the CG) were asked after the intervention phase (at T2) to specify the frequency of use of the bottles during the intervention period from 0 (very little use) to 6 (very much use). The OPR^++^ group was also asked to specify the frequency of the use of the audio file during the intervention period also from 0 (very little use) to 6 (very much use) at T2.

#### Data Analyses

We analyzed the differences between the physical as well as their mental well-being of the participants as the dependent variables, conducting different ANOVAs with Time as the repeated measures factor and Group (CG, OPR^–^, OPR^+^, OPR^++^) as the between-subjects factor. Bonferroni-corrected pairwise comparisons were used to follow up significant main effects (all pairwise comparisons had an adjusted alpha of 0.013). For the FEW-16, we investigated the total score of this questionnaire as well as the scores for the different subscales (*Resilience*, *Ability to Enjoy*, *Vitality*, and *Inner Peace)*. For the ARSS, we investigated the following subscales: *Physical Performance Capability*, *Mental Performance Capability*, *Emotional Balance*, *Overall Recovery*, *Muscular Stress*, *Lack of Activation*, *Negative Emotional State*, and *Overall Stress*.

### Results

#### Questionnaire for Assessing Subjective Physical Well-Being

For the FEW-16 total score, we found a significant main effect for Time, *F*(1, 64) = 9.199, *p* = 0.003, η^2^ = 0.126, with higher values at T2 compared to T1. The total score did not differ significantly between the four groups, *F*(3, 64) = 1.224, *p* = 0.308, η^2^ = 0.054. The interaction between Time × Group was not significant, *F*(3, 64) = 1.768, *p* = 0.162, η^2^ = 0.077.

Averaged across all groups, there was a main effect for the factor Time regarding the subscales *Vitality*, *F*(1, 64) = 9.117, *p* = 0.004, η^2^ = 0.125, and *Inner Peace*, *F*(1, 64) = 8,761, *p* = 0.004, η^2^ = 0.120, with higher values at T2 compared to T1, but not for the subscales *Resilience*, *F*(1, 64) = 0.028, *p* = 0.868, η^2^ < 0.001, and *Ability to Enjoy*, *F*(1, 64) = 2.555, *p* = 0.115, η^2^ = 0.038.

We did not find a significant Group effect for any of the subscales [*Vitality*: *F*(3, 64) = 1.964, *p* = 0.128, η^2^ = 0.084; *Resilience*: *F*(3, 64) = 0.637, *p* = 0.594, η^2^ = 0.029; *Inner Peace*, *F*(3, 64) = 0,967, *p* = 0.414, η^2^ = 0.043; *Ability to Enjoy*, *F*(3, 64) = 0.408, *p* = 0.748, η^2^ = 0.019].

**FIGURE 1:**
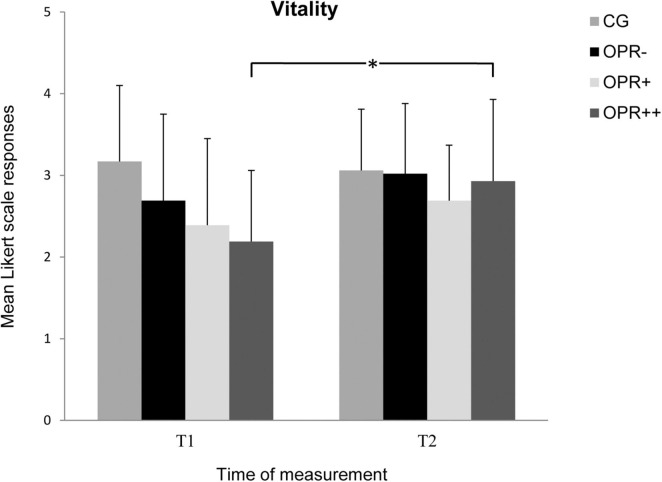
The mean values of the *Vitality* of the groups as a function of the time of measurement (T1, T2). The symbols represent across-participant means, and error bars show standard deviations. The 6-point Likert scale ranged from “does not apply at all” (0) to “fully applies” (5). (CG: control group without any intervention; OPR^–^ group: got an empty bottle for filling without any label on it; OPR^+^ group: got an empty bottle for filling with the label “Imagine I Am Health”; OPR^++^ group: got an empty bottle for filling with the label “Imagine I Am Health” and an audio file with a health mediation) (*p < 0.013).

Regarding the subscales, the interaction between Time × Group was significant for the subscale *Vitality*, *F*(3, 64) = 2.773, *p* = 0.049, η^2^ = 0.115 (Figure 1). Bonferroni-corrected pairwise comparisons showed significantly increased values from T1 to T2 for the OPR^++^ group (*p* = 0.007), with no other significant differences between T1 and T2 for any other group (*p* > 0.013). The Time × Group interaction was neither significant for *Inner Peace*, *F*(3, 64) = 1.967, *p* = 0.128, η^2^ = 0.084, *Resilience*, *F*(3, 64) = 8.50, *p* = 0.472, η^2^ = 0.038, nor for *Ability to Enjoy*, *F*(3, 64) = 0.355, *p* = 0.786, η^2^ = 0.016.

#### Acute Recovery and Stress Scale

With regard to the subscale *Physical Performance Capability*, the data revealed a significant main effect for the factor Time, *F*(1, 64) = 5.904, *p* = 0.018, η^2^ = 0.084. There was neither a Group effect, *F*(3, 64) = 0.386, *p* = 0.764, η^2^ = 0.018, nor the interaction between Group × Time was significant, *F*(3, 64) = 1.118, *p* = 0.348, η^2^ = 0.050.

On the other *Recovery* scales in the ARSS, we found no significant effect of Time for the subscales *Mental Performance Capability* (*p* = *0.529*), *Emotional Balance* (*p* = 0.107), and *Overall Recovery* (*p* = 0.073). Furthermore, there were neither any Group effect for these three subscales (*Mental Performance Capability*: *p* = 0.913; *Emotional Balance*: *p* = 0.525; *Overall Recovery*: *p* = 0.819) nor any significant interactions between Time and Group (*Mental Performance Capability*: *p* = 0.340; *Emotional Balance*: *p* = 0.993; *Overall Recovery*: *p* = 0.595).

Looking at the *Stress* dimension in the ARSS, we found a significant effect of Time for the subscale *Muscular Stress*, *F*(1, 64) = 4.314, *p* = 0.042, η^2^ = 0.063, but not for *Lack of Activation* (*p* = 0.192), *Negative Emotional State* (*p* = 0.520) and *Overall Stress* (*p* = 0.288). There was neither a significant Group effect for any of these stress subscales (*Muscular Stress*: *p* = 0.965; *Lack of Activation*: *p* = 0.598; *Negative Emotional State*: *p* = 0.205; *Overall Stress*: *p* = 0.956) nor a significant interaction between Time and Group (*Muscular Stress*: *p* = 0.184; *Lack of Activation*: *p* = 0.979; *Negative Emotional State*: *p* = 0.765; *Overall Stress*: *p* = 0.397).

All the *p*-values and effect sizes for the interactions among Group (CG, OPR^–^, OPR^+^, OPR^++^) and Time (T1, T2) calculated by the use of the ANOVAs regarding the FEW-16 and ARSS subscales are summarized in Table 1.

**TABLE 1:** Interaction relationships for Group (CG, OPR^–^, OPR^+^, OPR^++^) and Time (T1, T2) as predictors of the subscales in the FEW-16 and ARSS (Experiment 1).

**Dependent variable**	**P**	**η _p_^2^**
FEW-16 (total score)	0.162	0.077
FEW-16 (subscale vitality)	**0.049**	**0.115**
FEW-16 (subscale inner peace)	0.128	0.084
FEW-16 (subscale resilience)	0.472	0.038
FEW-16 (subscale ability to enjoy)	0.786	0.016
ARSS (physical performance capability)	0.348	0.050
ARSS (mental performance capability)	0.340	0.051
ARSS (emotional balance)	0.993	0.001
ARSS (overall recovery)	0.595	0.029
ARSS (muscular stress)	0.184	0.072
ARSS (lack of activation)	0.979	0.003
ARSS (negative emotional state)	0.765	0.018
ARSS (overall stress)	0.397	0.045

*Significant results are in bold.*

#### Use of Water Bottles and Audio File

The participants in the OPR^–^ group rated the use of the specially assigned bottles with 4.50 (*SD* = 1.50) on the 6-point Likert scale, the OPR^+^ group with *M* = 4.28 (*SD* = 1.22), and the OPR^++^ group with *M* = 4.76 (*SD* = 1.03). There were no significant differences between these three groups with regard to the use of their assigned bottles (*p* > 0.05). The OPR^++^ group was also asked to mention the frequency of use of the audio file during the intervention period and the use of the audio file was evaluated with *M* = 3.00 (*SD* = 1.83) on the Likert scale (0 – very less use to 6 – very much use).

### Discussion

The results of Experiment 1 could not fully confirm our first hypothesis that the well-being of the participants is enhanced with the placebo using the water with a rationale and the label “Imagine I am Health” (OPR^+^ OPR^++^) compared with the OPR^–^ group and the CG. However, even though most of the Time × Group interactions missed significance, we could observe some tendencies that the rationale combined with our labeled water (OPR^+^ OPR^++^) improved the well-being of the participants from T1 to T2. The results could also not fully confirm our second hypothesis that the positive effect on the well-being the participants is enhanced when adding an additional rationale (through an audio file) in a relaxed, suggestive state (OPR^++^) compared with just a rationale in an awake state (OPR^+^). Almost all comparisons missed significance. Only the subscale *Vitality* showed a significant interaction effect between the time of measurement and the factor Group. The OPR^++^ group showed an increasing *Vitality* score from T1 to T2, indicating that the labeled bottle in combination with an additional rationale in a relaxed suggestive state had a positive effect on the well-being of the participants. In general, subjective vitality is described as a relevant measure of subjectively experienced positive psychological well-being (Rouse et al., 2015), a state being associated with the feeling of being alive, vital, and full of energy (Ryan and Frederick, 1997). In summary, in Experiment 1, we could not find adequate support that our specific kind of OLP intervention with just one (refillable) water bottle worked well in the OPR^+^ or the OPR^++^ group. This could be due to a possible flaw in the design that the experimental groups got only one filled bottle of water and were asked to refill this bottle themselves as soon as it was empty. It might be assumed that the anticipated placebo effect decreased over time, in particular, when the participants had to refill the bottle by themselves. Even before executing Experiment 1, we took into account the idea that the participants’ self-refilling of the labeled bottles might decrease the possible effects. Therefore, before starting Experiment 1, we decided to also implement Experiment 2 with the modified design where participants had a fresh sealed water bottle every day with the label “Imagine I am Health” in the OPR^+^ and the OPR^++^ group.

## Experiment 2

After a 2-week intervention period, Experiment 1 revealed a positive effect on vitality for individuals who consumed the placebo water and got an additional rationale in a relaxed suggestive state (OPR^++^). Experiment 2 was designed in parallel to replicate that finding and to explore further whether the state of well-being can be increased if the participants not only got an empty bottle of water to refill, but rather get a full bottle of water daily during the 2-week intervention period. In particular, we predicted that the tendencies which were expected in Experiment 1 should be more obvious in Experiment 2—more specifically, we assumed that the participants getting a fresh bottle of water every day (OPR^+^, OPR^++^) would feel better and healthier compared to an OPR^–^ group and a CG and that the participants who got an additional rationale with the audio file (OPR^++^ group) would improve their subjective health status even more.

### Method

#### Participants

Power analyses again indicated a sample size of at least 56 participants. Eighty participants (32 males, 48 females) aged 18–83 years (*M*_age_ = 26.51 years, *SD* = 9.94 years) took part at the beginning of Experiment 2. However, five of these participants dropped out during the intervention, so we finally analyzed the data of 75 participants. Informed consent in accordance with the Declaration of Helsinki was obtained from each participant prior to testing and ethical approval was again obtained from the lead institution (number 171/2020).

#### Procedure

The participants were randomly assigned to a CG (*n* = 18), an OPR^–^ (*n* = 19), an OPR^+^ (*n* = 19), and an OPR^++^ (*n* = 19) group. We used the same 2 × 4 design and also the same materials as in Experiment 1. The only difference was that all the participants besides the CG got not only one bottle to refill (like in Experiment 1), but every day during the intervention period, they received a new sealed bottle of water. All the participants, besides the CG, received all the bottles for the intervention phase at T1 after filling out the questionnaires, so there were no further interactions with the investigator.

### Results

#### Questionnaire for Assessing Subjective Physical Well-Being

For the FEW-16 total score, we found no significant main effect for the factor Group, *F*(3, 71) = 1.409, *p* = 0.247, η^2^ = 0.056, but for the factor Time, there was an observable effect *F*(1, 71) = 37.656, *p* = 0.000, η^2^ = 0.347, indicating that the score increased from T1 to T2. The interaction Time × Group failed significance, *F*(3, 71) = 0.704, *p* = 0.553, η^2^ = 0.029.

For the subscale *Resilience*, we found a significant main effect for the factor Time, *F*(1, 71) = 3.706, *p* = 0.058, η^2^ = 0.050. Group values did not differ significantly, *F*(3, 71) = 1.288, *p* = 0.285, η^2^ = 0.052. There was no significant interaction between Time and Group, *F*(3, 71) = 0.437, *p* = 0.727, η^2^ = 0.018.

Regarding the subscale for *Ability to Enjoy*, a repeated measure ANOVA revealed a main effect for the factor Time, *F*(1, 71) = 9.592, *p* = 0.003, η^2^ = 0.119, indicating that the scores were higher at T2 than at T1. There was neither a significant Group effect, *F*(3, 71) = 0.705, *p* = 0.552, η^2^ = 0.029, nor a Time × Group interaction effect, *F*(3, 71) = 0.712, *p* = 0.548, η^2^ = 0.029.

We found a main effect for the factor Time concerning the subscale *Vitality*, *F*(1, 71) = 32.050, *p* < 0.001, η^2^ = 0.311, indicating that the scores were significantly higher at T2 compared to T1. There was also a significant Group effect, *F*(3, 71) = 4.517, *p* = 0.006, η^2^ = 0.160: Bonferroni-corrected pairwise comparisons showed higher values for the CG than for the OPR^+^ group (*p* = 0.044), and the OPR^++^ group reported higher scores than the OPR^+^ group (*p* = 0.017), with no differences between the other group comparisons (*p* > 0.013). Moreover, the interaction Time × Group was significant, *F*(3, 71) = 3.795, *p* = 0.014, η^2^ = 0.138: Bonferroni-corrected pairwise analyses showed differences between T1 and T2 for the OPR^–^ group with higher scores for T2 compared to T1 (*p* < 0.001), but not for the CG (*p* = 0.098), the OPR^+^ (*p* = 0.025), and the OPR^++^ groups (*p* = 0.159).

Furthermore, we found a significant main effect for the factor Time for *Inner Peace*, *F*(1, 71) = 16.983, *p* < 0.001, η^2^ = 0.193, again showing higher scores at T2 compared to T1 across all groups. There was no Group effect, *F*(3, 71) = 0.780, *p* = 0.509, η^2^ = 0.032, and no significant interaction between Time and Group, *F*(3, 71) = 1.210, *p* = 0.312, η^2^ = 0.049.

#### Acute Recovery and Stress Scale

**FIGURE 2:**
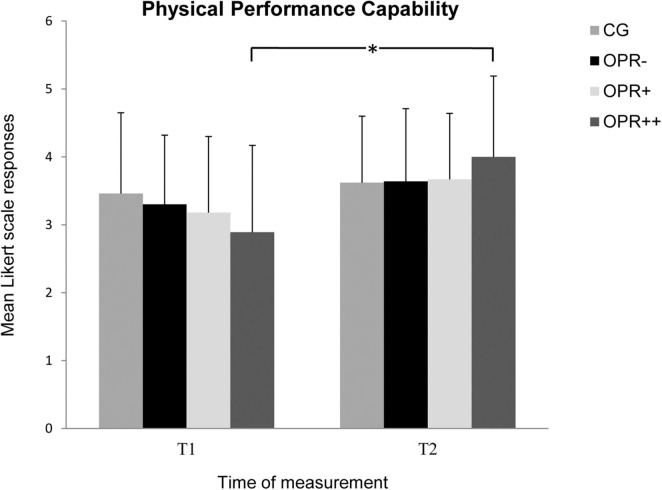
The mean values of the *Physical Performance Capability* of the groups as a function of time of measurement (T1, T2). The symbols represent across-participant means, and error bars show standard deviations. The 7-point Likert scale ranged from “does not apply at all” (0) to “fully applies” (6). (CG: control group without any intervention; OPR^–^ group: got a filled bottle of water every day without any label on it; OPR^+^ group: got a filled bottle of water every day with the label “Imagine I Am Health”; OPR^++^ group: got a filled bottle of water every day with the label “Imagine I Am Health” and an audio file with a health mediation) (*p < 0.013).

On the subscale *Physical Performance Capability*, the data revealed a significant main effect for the factor Time, *F*(1, 71) = 18.919, *p* < 0.001, η^2^ = 0.210, but not for Group, *F*(3, 71) = 0.049, *p* = 0.986, η^2^ = 0.002. The interaction between Group × Time was significant, *F*(3, 71) = 2.961, *p* = 0.038, η^2^ = 0.111. Follow-up Bonferroni-adjusted pairwise comparisons showed that the participants in all four groups reported higher physical performance capability from T1 to T2, but only the scores of the OPR^++^ group (*p* = 0.002) differed significantly between both the time measurements, with no significant differences for the other three groups (CG: *p* = 0.514; OPR^–^: *p* = 0.025; OPR^+^: *p* = 0.065; see Figure 2).

**FIGURE 3:**
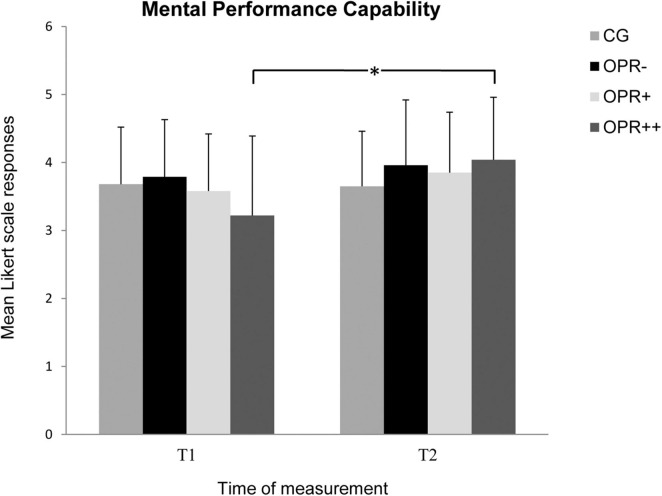
The mean values of the *Mental Performance Capability* of the groups as a function of time of measurement (T1, T2). The symbols represent across-participant means, and error bars show standard deviations. The 7-point Likert scale ranged from “does not apply at all” (0) to “fully applies” (6). (CG: control group without any intervention; OPR^–^ group: got a filled bottle of water every day without any label on it; OPR^+^ group: got a filled bottle of water every day with the label “Imagine I Am Health”; OPR^++^ group: got a filled bottle of water every day with the label “Imagine I Am Health” and an audio file with a health mediation) (*p < 0.013).

We did not find a significant Group effect for the subscale *Mental Performance Capability*, *F*(3, 71) = 0.347, *p* = 0.792, η^2^ = 0.014, but there was an observable effect for the factor Time, *F*(1, 71) = 8.587, *p* = 0.005, η^2^ = 0.108. Furthermore, the interaction between Time and Group was significant, *F*(3, 71) = 3.158, *p* = 0.030, η^2^ = 0.118 (see Figure 3). Follow-up Bonferroni-corrected pairwise comparisons showed that participants in the OPR^++^ group reported a significant higher *Mental Performance Capability* from T1 to T2 (*p* = 0.005) in contrast to the CG (*p* = 0.807), the OPR^–^ (*p* = 0.256), and the OPR^+^ (*p* = 0.307) group.

**FIGURE 4:**
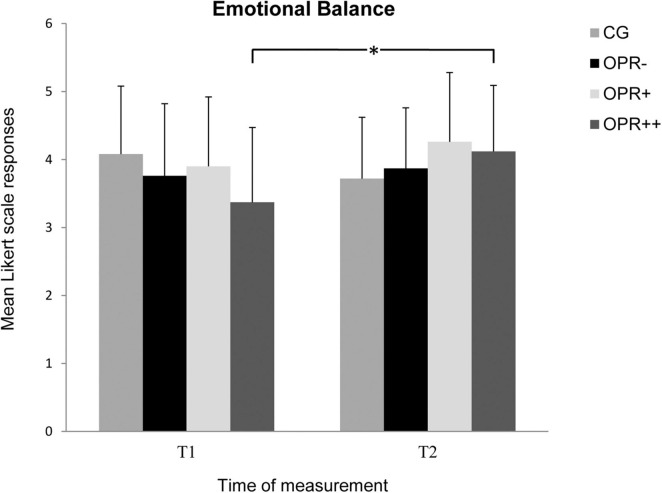
The mean values of the *Emotional Balance* of the groups as a function of the time of measurement (T1, T2). The symbols represent across-participant means, and error bars show standard deviations. The 7-point Likert scale ranged from “does not apply at all” (0) to “fully applies” (6). (CG: control group without any intervention; OPR^–^ group: got a filled bottle of water every day without any label on it; OPR^+^ group: got a filled bottle of water every day with the label “Imagine I Am Health”; OPR^++^ group: got a filled bottle of water every day with the label “Imagine I Am Health” and an audio file with a health mediation) (*p < 0.013).

Analyses for the subscale *Emotional Balance* showed no significant effect for the factor Group, *F*(3, 71) = 0.503, *p* = 0.682, η^2^ = 0.021, but did for the factor Time, *F*(3, 71) = 4.182, *p* = 0.045, η^2^ = 0.056. The results showed higher scores again at T2 compared to T1 across all groups. Furthermore, there was a significant interaction between Time and Group, *F*(3, 71) = 4.907, *p* = 0.004, η^2^ = 0.172 (see Figure 4). The scores significantly increased from T1 to T2 for the OPR^++^ group (*p* = 0.009), but not for the three other groups (CG: *p* = 0.014; OPR^–^: *p* = 0.617; OPR^+^: *p* = 0.133).

**FIGURE 5:**
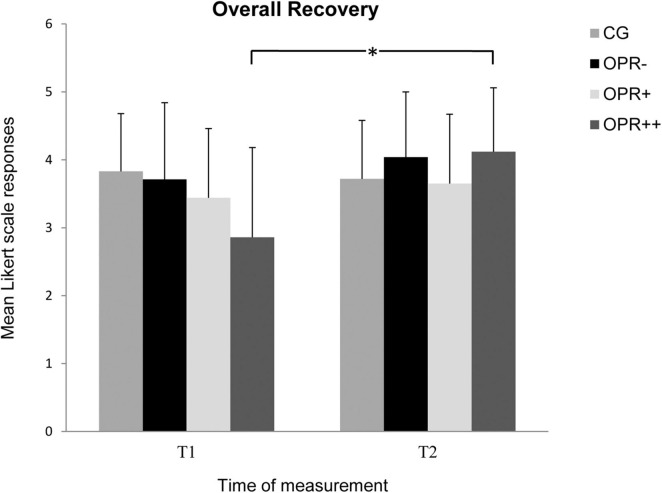
The mean values of the *Overall Recovery* of the groups as a function of the time of measurement (T1, T2). The symbols represent across-participant means, and error bars show standard deviations. The 7-point Likert scale ranged from “does not apply at all” (0) to “fully applies” (6). (CG: control group without any intervention; OPR^–^ group: got a filled bottle of water every day without any label on it; OPR^+^ group: got a filled bottle of water every day with the label “Imagine I Am Health”; OPR^++^ group: got a filled bottle of water every day with the label “Imagine I Am Health” and an audio file with a health mediation) (*p < 0.013).

For the subscale *Overall Recovery*, we found no main effect for Group, *F*(3, 71) = 0.736, *p* = 0.534, η^2^ = 0.030, but for Time, *F*(1, 71) = 18.738, *p* < 0.001, η^2^ = 0.209. Furthermore, there was a significant interaction between Time and Group, *F*(3, 71) = 9.144, *p* < 0.001, η^2^ = 0.279 (see Figure 5). Follow-up Bonferroni correcting pairwise comparisons showed that the participants of the OPR^++^ Group reported a significant higher *Overall Recovery* from T1 to T2 (*p* < 0.001) in contrast to all the other groups (CG: *p* = 0.373; OPR^–^ *p* = 0.135; OPR^+^: *p* = 0.209).

**FIGURE 6:**
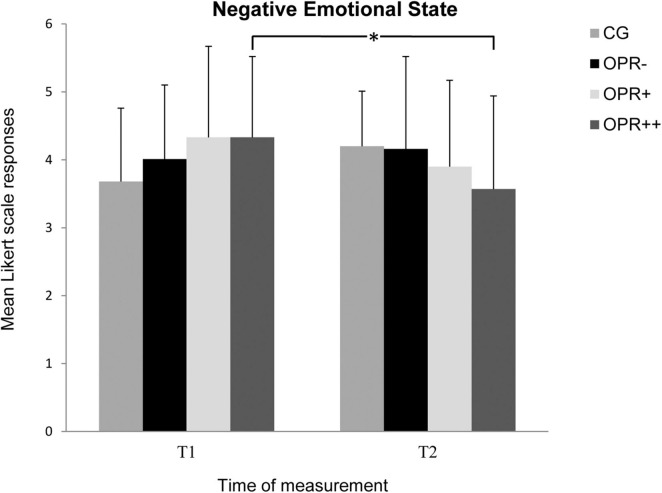
The mean values of the *Negative Emotional State* of the groups as a function of the time of measurement (T1, T2). The symbols represent across-participant means, and error bars show standard deviations. The 7-point Likert scale ranged from “does not apply at all” (0) to “fully applies” (6). (CG: control group without any intervention; OPR^–^ group: got a filled bottle of water every day without any label on it; OPR^+^ group: got a filled bottle of water every day with the label “Imagine I Am Health”; OPR^++^ group: got a filled bottle of water every day with the label “Imagine I Am Health” and an audio file with a health mediation) (*p < 0.013).

**FIGURE 7:**
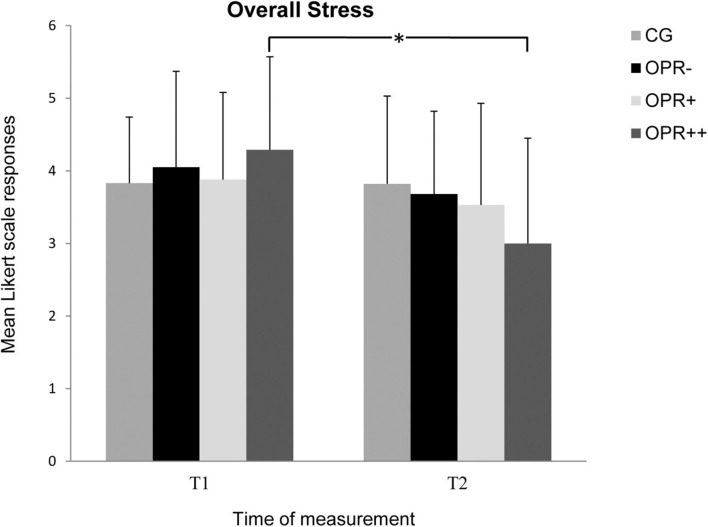
The mean values of the *Overall Stress* of the groups as a function of the time of measurement (T1, T2). The symbols represent across-participant means, and error bars show standard deviations. The 7-point Likert scale ranged from “does not apply at all” (0) to “fully applies” (6). (CG: control group without any intervention; OPR^–^ group: got a filled bottle of water every day without any label on it; OPR^+^ group: got a filled bottle of water every day with the label “Imagine I Am Health”; OPR^++^ group: got a filled bottle of water every day with the label “Imagine I Am Health” and an audio file with a health mediation) (*p < 0.013).

Regarding the Stress Scales in the ARSS, there was no Group effect for any of the subscales *Muscular Stress*, *Lack of Activation*, *Negative Emotional State*, and *Overall Stress* (*p* > 0.05). Furthermore, there was no significant Time effect for the subscales *Muscular Stress* and *Negative Emotional State* (*p* > 0.05), but it was observed for the subscales *Lack of Activation*, *F*(1, 71) = 7.577, *p* = 0.007, η^2^ = 0.096, and *Overall Stress*, *F*(1, 71) = 12.719, *p* = 0.001, η^2^ = 0.152. The interactions between Time and Group failed significance for Muscular Stress and *Lack of Activation* (*p* > 0.05), but not for *Negative Emotional State*, *F*(3, 71) = 4.654, *p* = 0.005, η^2^ = 0.164 (Figure 6), and *Overall Stress*, *F*(3, 71) = 3.797, *p* = 0.014, η^2^ = 0.138 (Figure 7). For *Negative Emotional State*, the Bonferroni-corrected pairwise analyses showed differences between T1 and T2 for the OPR^++^ group (*p* = 0.012), but not for the CG (*p* = 0.024), the OPR^–^ group (*p* = 0.626), or the OPR^+^ group (*p* = 0.148). For *Overall Stress*, the Bonferroni-corrected pairwise analyses again showed differences between T1 and T2 for the OPR^++^ group (*p* = 0.001), but not for the CG (*p* = 0.964), the OPR^–^ group (*p* = 0.224), or the OPR^+^ group (*p* = 0.147).

All the *p*-values and effect sizes for the interactions among Group (CG, OPR^–^, OPR^+^, OPR^++^) and Time (T1, T2) calculated by the use of the ANOVAs regarding the FEW-16 and ARSS subscales are summarized in Table 2.

**TABLE 2:** Interaction relationships for Group (CG, OPR^–^, OPR^+^, OPR^++^) and Time (T1, T2) as predictors of the subscales in the FEW-16 and ARSS (Experiment 2).

**Dependent variable**	**p**	**η _p_^2^**
FEW-16 (total score)	0.553	0.029
FEW-16 (subscale vitality)	**0.014**	**0.138**
FEW-16 (subscale inner peace)	0.312	0.049
FEW-16 (subscale resilience)	0.727	0.018
FEW-16 (subscale ability to enjoy)	0.548	0.029
ARSS (physical performance capability)	**0.038**	**0.111**
ARSS (mental performance capability)	**0.030**	**0.118**
ARSS (emotional balance)	**0.004**	**0.172**
ARSS (overall recovery)	** < 0.001**	**0.279**
ARSS (muscular stress)	0.374	0.043
ARSS (lack of activation)	0.188	0.065
ARSS (negative emotional state)	**0.005**	**0.164**
ARSS (overall stress)	**0.014**	**0.138**

*Significant results are in bold.*

#### Use of Water Bottles and Audio File

The participants in the OPR^–^ Group rated the use of the specifically assigned bottles on a Likert scale from 0 (very little use) to 6 (very much use) with *M* = 4.95 (*SD* = 1.10), the OPR^+^ Group with *M* = 4.00 (*SD* = 1.20), and OPR^++^ with *M* = 4.74 (*SD* = 1.29). There were no significance differences between these three groups in the way they used their specific bottles. For the audio file in OPR^++^ group, the total usage on a Likert scale 0 (very little use) to 6 (very much use) was assessed with *M* = 3.05 (*SD* = 1.10).

### Discussion

The results of Experiment 2 also showed a significant interaction effect between the time of measurement and the factor Group for the subscale *Vitality* in the FEW-16 as was in Experiment 1. Although, the values in the OPR^+^ and OPR^++^ groups increased, just like in Experiment 1, from T1 to T2, the only significant improvement in the *Vitality* score from T1 to T2 was surprisingly observed for the OPR^–^ group with the unlabeled bottle. A possible explanation for the enhancement in these two variables might be that OPR^–^ group showed the most frequent use of the water bottles of all the groups.

In the ARSS, we found significant interactions in all the recovery scales (*Physical Performance*, *Mental Performance*, *Emotional Balance*, and *Overall Recovery*) similar to those in the two stress scales (*Negative Emotional State* and *Overall Stress*). Especially the additional rationale in a trance state group increased the participants’ values in all the recovery scales significantly and decreased their values significantly in two of the four stress subscales. Therefore, we only found some latent tendencies that awareness of the rationale in an awake state combined with our labeled bottles themselves (OPR^+^) had a positive impact on the well-being of the participants. However, additional rationale in a trance state in addition to a rationale in an awake state (OPR^++^) had a more positive effect on the well-being of the participants. This combination in OPR^++^ seemed to increase the recovery state of the participants and reduce their stress level.

## General Discussion

Over the last few decades, administration of placebos has indicated positive effects not only when the subjects received mock drugs or mock agents as deceptive placebos, i.e., when they were not aware about the ineffectiveness of the medications, but also when they were aware of the ineffectiveness of the substances, i.e., when they received OLPs (e.g., Kam-Hansen et al., 2014; Carvalho et al., 2016; Schaefer et al., 2016). However, so far, it was unclear whether positive effects can only be accomplished by medication or routinely used products may also lead to improved well-being and an improved health status. Previous studies have shown that dehydration can lead to decreased cognitive performance (Grandjean and Grandjean, 2007; Adan, 2012) and physical functioning (Baker et al., 2007). Our study aimed to analyze the effects of OLPs when subjects used a commonly used product.

In two experiments, the participants were randomly assigned to different groups with differing conditions with regard to receiving water bottles with a rationale they received from the investigator, either in an awake (OPR^+^) state or in an awake plus trance state (OPR^++^). In Experiment 1, most of the interactions for the well-being and health status of the participants failed (barely) significance with the exception of the subscale *Vitality*. The FEW-16 which was used in the current study is a validated questionnaire to assess the general and mental well-being of people (Lashki et al., 2017). Regarding the health and well-being state of the participants, we could only find in Experiment 1 that the reported values of the groups for the subscale *Vitality* in the FEW16 varied as a function of the time of measurement. In contrast to Experiment 1, we could find several significant Time × Group interactions in Experiment 2. We also found that the reported values of the different groups for the subscale *Vitality* in the FEW-16 differed as a function of the time of measurement. The ARSS was also used to assess different dimensions of recovery and stress. The reported group values varied as a function of the time of measurement for the subscales *Physical Performance Capability*, *Mental Performance Capability*, *Emotional Balance*, *Overall Recovery*, *Negative Emotional State*, and *Overall Stress*. All of the pairwise tests in the mentioned subscales showed similar pattern of results: OPR^+^ reported higher values in the mentioned recovery scales and lower values in the stress scales, but only for OPR^++^, the improvements reached significance. So, we could not fully confirm our hypothesis that the rationale in an awake state itself leads to a significant enhanced subjective health state. For the best effect on subjective well-being, it seems necessary to combine the rationale with an additional rationale in a relaxed trance state. Thus, these group members demonstrated an OLP effect—although, they knew that they were taking a placebo. Our findings complement current research of open-label placebo effectiveness. While studies so far have emphasized the fundamental role of a rationale (e.g., Locher et al., 2017), we could show that the effectiveness of a convincing rationale provided by an investigator can be successfully supported by an additional rationale in a trance state.

Our experiments also allude to the tendency that OLP^++^ in the form of water with health claims may be more effective when the water is given in sealed bottles than in a refillable bottle. A possible explanation might be that OLP effects are more pronounced when the stimulus is more external (every day a new sealed bottle) without an active help of the participants (refilling of the bottle).

There are some limitations and considerations for future research that need to be acknowledged. The allocation of the participants to four different groups was not obscured and, therefore, there was no blind outcome assessment in our experiments. Furthermore, the participants were given a pre- and a post-test survey to answer, but not a retention test some days or weeks after the intervention. Therefore, we can only conclude with the current results that an OLP intervention with drinking water has some positive effects on subjective physical and mental well-being directly following the intervention, but we cannot say anything about its sustainability. Furthermore, in order to find out the use of the specifically assigned water bottles to the participants of the OPR^–^, OPR^+^, and OPR^++^ groups as well as the use of the audio file that was given to OPR^++^ group, after the intervention period, the participants were asked to specify the frequency of the use of the bottles and audio file during the last 2 weeks on Likert scales ranging from 0 (very little use) to 6 (very much use). Since an estimation and an exact reproduction after a period of 2 weeks are error-prone, future research should ask participants to use a diary in which they have to note their individual use of the bottles as well as the use of the audio file at the end of each day. Regarding the OPR^++^ group, future studies should investigate the effect of additional rationales in a relaxed trance state on the well-being and reported health status of the participants in more detail. In Experiment 2, OPR^++^ participants reported a significant increase of their well-being and an improved health status more often than the other groups. This might be a major design issue because it is likely that the well-being effects could be attributed only to the trance state. Our assumption is that our specific design where people got an external stimulus (one closed fresh bottle every day) in combination with another rationale in a trance state might be responsible for the results in Experiment 2 for the OPR^++^ group. However, we cannot conclusively say that it was the combination of the rationale in an awake state and the additional rationale in a relaxed trance state that had the most influence on the outcome of the experiment and whether it was the relaxation/hypnotic suggestions in the trance state that was most responsible for the increased values of the reported well-being of the participants. We recommend that further studies could include a relaxation (trance state) without placebo condition to test this empirically. Furthermore, all the groups, except the control group, were aware of the purpose of the study. If the participants in the control group had been made aware of the purpose of the study, their expectations might have been elevated and this could have possibly understated the results due to interference.

There is an increasing number of studies suggesting that OLPs are effective in alleviating symptoms (Kelley et al., 2012; Carvalho et al., 2016; Schaefer et al., 2016). The effects of OLPs have been often explained by the same mechanism as by placebos, namely, associative learning or conscious expectations (Charlesworth et al., 2017). Individuals receiving OLPs certainly do not have the same level of conscious expectations as those receiving deceptive placebos; however, OLPs are most commonly combined with positive recommendations. This was also the case in the current study with water bottles with a rationale including the label “Imagine I Am Health.” This statement was likely associated with the assumption that drinking water from this bottle is effective and health-promoting, and therefore triggering a positive expectancy—a top-down mechanism. It would be interesting to investigate the effects of different labels on the bottles in future research and to investigate whether people are also positively influenced by terms other than “Health,” such as “Happiness” or “Energy.” Moreover, it should be examined if people are not only influenced by positive labels but also by negative ones. There is already some evidence, at least in the medical research, that negative expectations, for example, adversely affect health, most often by increasing pain perception (Bingel et al., 2011).

For over 200 years, there are debates over the ethical use of placebos, especially in clinical practice (e.g., Jutte, 2013). The key aspect is always that the placebo effect necessitates subjects (in this case often patients) being unaware of being treated with a physiologically inert substance and the resulting deception. In contrast, in OLP studies, participants are explicitly informed in advance that they will receive a placebo before evaluating the effectiveness of the placebo intervention. Empirical findings to date tentatively support the issue that subjects consider OLPs to be ethical (Blease et al., 2016).

Open-label placebos can lead to relevant changes in the subjective experiences in healthy participants; however, the critical factor here is the form of rationale which effectuates the meaning (Locher et al., 2017). Our study shows that OLP in the form of water with a rationale might be a promising indicator that a commonly used product can have additional benefits for personal well-being. However, we found the tendency that this effect can only be observed with the rationale in a trance state and more with freshly labeled bottles than with refillable ones. Our findings open the doors for a multitude of follow-up research on the use of OLPs. Eventually, this can be used in a variety of fields to complement traditional interventions along with OLPs to maximize the recovery of individuals.

## Data Availability Statement

The raw data supporting the conclusions of this article will be made available by MR (m.rathschlag@dshs-koeln.de), without undue reservation, to any qualified researcher.

## Ethics Statement

The studies involving human participants were reviewed and approved by the Ethics Committee of the German Sport University Cologne. The patients/participants provided their written informed consent to participate in this study.

## Author Contributions

Both authors listed have made a substantial, direct, and intellectual contribution to the work, and approved it for publication.

## Conflict of Interest

The authors declare that the research was conducted in the absence of any commercial or financial relationships that could be construed as a potential conflict of interest.

## Publisher’s Note

All claims expressed in this article are solely those of the authors and do not necessarily represent those of their affiliated organizations, or those of the publisher, the editors and the reviewers. Any product that may be evaluated in this article, or claim that may be made by its manufacturer, is not guaranteed or endorsed by the publisher.
